# Antimycobacterial Activity of Essential Oils from Bulgarian *Rosa* Species Against Phylogenomically Different *Mycobacterium tuberculosis* Strains

**DOI:** 10.3390/pharmaceutics16111393

**Published:** 2024-10-29

**Authors:** Violeta Valcheva, Milka Mileva, Marine Dogonadze, Ana Dobreva, Igor Mokrousov

**Affiliations:** 1 The Stephan Angeloff Institute of Microbiology, Bulgarian Academy of Sciences, 26 Acad. G. Bonchev Str., 1113 Sofia, Bulgaria; milkamileva@gmail.com; 2 Laboratory of Microbiology, Biochemistry and Immunogenetics, St. Petersburg Research Institute of Phthisiopulmonology, 2-4 Ligovsky Prospect, 191036 St. Petersburg, Russia; marine-md@mail.ru; 3 Institute for Roses and Aromatic Plants, Agricultural Academy, 49 Osvobojdenie Blvd., 6100 Kazanlak, Bulgaria; anadobreva@abv.bg; 4 Laboratory of Molecular Epidemiology and Evolutionary Genetics, St. Petersburg Pasteur Institute, 14 Mira Str., 197101 St. Petersburg, Russia; 5 Henan International Joint Laboratory of Children’s Infectious Diseases, Children’s Hospital Affiliated to Zhengzhou University, Henan Children’s Hospital Zhengzhou Children’s Hospital, Zhengzhou 450012, China

**Keywords:** *Mycobacterium tuberculosis*, *Rosa damascena* Mill., *Rosa alba* L., *Rosa centifolia* L., *Rosa gallica* L., essential oils

## Abstract

In this study, we aimed to assess the activity of the essential oils from four Bulgarian oil-bearing roses *Rosa damascena* Mill., *R. alba* L., *R. centifolia* L., and *R. gallica* L., on the reference strain *Mycobacterium tuberculosis* H37Rv and clinical *M. tuberculosis* strains of the Beijing and Latin-American Mediterraneum genotypes. The chemical composition of the essential oils was determined by gas chromatography (GC-FID/MS). Minimal inhibitory concentrations (MIC) were determined using the resazurin method. *R. alba* oil showed the highest inhibitory activity when tested on all strains of different phylogenetic origins with MIC in the range of 0.16–0.31 mg/mL, while *R. gallica* oil was the least active (MIC 0.62–1.25 mg/mL). The obtained results show heterogeneity of rose oil action on different mycobacterial strains and we hypothesize that the combined level of geraniol and nerol is a key factor that underlies the antimycobacterial action of the rose oils. Strain Beijing 396 was relatively more susceptible to the rose oils probably due to multiple and likely deleterious mutations in its efflux pump genes. Two clinical MDR strains have likely developed during their previous adaptation to anti-TB drugs certain drug tolerance mechanisms that also permitted them to demonstrate intrinsic tolerance to the essential oils. Further research should investigate a possible synergistic action of the new-generation anti-TB drugs and the most promising rose oil extracts on the large panel of different strains.

## 1. Introduction

Tuberculosis (TB) is an infectious disease caused by *Mycobacterium tuberculosis* and presents a global health concern with a significant impact on the worldwide economy. The drugs and vaccines developed against tuberculosis, as well as therapies and preventive measures, are insufficient to stop the dissemination of *M. tuberculosis* strains. With the increase in the migration processes in the last decades, a resurgence and spread of drug-resistant tuberculosis strains was described. The decreasing TB incidence rates in some high-burden countries are accompanied by increasing emergence and circulation of strains with multiple and extensive drug resistance [1,2,3].

The fight against drug resistance in the prevention and therapy of bacterial diseases has engaged the attention of many scientific teams. Successful therapeutic approaches are often based on the healing properties of plant extracts.

Plant extracts from oil-bearing plants are distinguished by excellent antibacterial properties because of the wide range of phytocomplexes and structurally diverse compounds contained in them [4]. Most of the medical properties of aromatic plants, including antibacterial action, are mainly attributed to their essential oils [5]. *Rosa damascena* Mill., *Rosa alba* L., *Rosa gallica* L., and *Rosa centifolia* L. are the main oil-bearing roses in Bulgaria, also called “old roses”, considered raw materials for the perfumery and cosmetics industries. The use of rose essential oils, not only as fragrances or aromatherapy but also for the medication of various diseases and disorders finds wide application in Bulgarian folk medicine [6,7,8]. Even in the works of Avicenna and other ancient authoritative medical books, there is information about the use of these natural products (named “Go-Langevin”/”Golqand”, “Jollab” and syrups) as a remedy for tuberculosis despite these effects have not been scientifically explained [9]. In the last few decades, empirical knowledge from folk medicine has been an important aspect of in vitro and in vivo research, including preclinical and clinical trials. High-valuable medicinal properties have been observed in roses, especially for their essential oils, including antidepressant effects [10,11,12], antioxidant [13,14,15], antimicrobial, antifungal [16], and probiotic [17] activity.

Rose oils are volatile, liquid, transparent, soluble in lipids, rarely colored, and well soluble in organic solvents [18]. An important characteristic of all essential oils and their components is their strong hydrophobicity. This allows them to adhere to lipids in the cell membranes of bacteria, which destroys cell structures and, as a result, increases membrane permeability. The likely outcome of this process is the death of a bacterial cell due to the leakage of critical molecules and ions from the cell contents. Rose essential oils deserve special attention also because of their valuable properties for capturing free radicals and controlling oxidative damage, which affect the integrity of biomolecules and hinder the cellular metabolic chains underlying the redox pathogenesis of the diseased organism in the course of viral and bacterial infections [18].

Lipophilic essential oils penetrate the phospholipid bilayer of cell membranes and lower their integrity, which explains their antimicrobial activity against bacteria and fungi [19].

The main ingredients of rose oil as monoterpene alcohols (including linalool, nerol, citronellol, and geraniol) were shown to be effective against bacteria rather than fungi [20]. Geraniol is an acyclic monoterpene alcohol common constituent of several essential oils including rose oil. The functional groups present in the active components of essential oils and their possible synergistic interactions contribute to their antibacterial activity [21,22]. The wide range of valuable biological properties of the essential oils from roses are due to terpene alcohols and hydrocarbons [23,24]. The main components of essential oil of Bulgarian roses are terpene alcohols geraniol (15.85–34.02%), citronellol (6.70–28.72%), and nerol (5.80–11.90%). Hydrocarbons are represented by saturated aliphatic homologs with an odd number of carbon atoms, the main ones being nonadecane (8.10–22.67%), heneicosane (4.37–10.21%), heptadecane (1.07–2.98%) and triclosan (0.81–5.90%).

The antimicrobial mechanism of action also depends on the studied microorganism. Gram-positive bacteria are more susceptible than Gram-negative bacteria to the anti-bacterial action of essential oil ingredients [25,26]. The outer membrane of gram-negative bacteria is rigid and rich in lipopolysaccharide, with a more complex structure that limits the diffusion of hydrophobic compounds. Gram-positive bacteria are enveloped by a thick peptidoglycan wall that is not dense enough to facilitate the access of small antimicrobial molecules [27]. Mycobacteria are characterized by unusually low cell wall permeability, which modulates the rapid development of resistance to therapeutic agents. The cell wall skeleton contains a large number of fatty acids, in particular, the mycolic acids, covalently bound to arabinogalactan. In the innermost part of the double lipid bilayer, fluidity is low, increasing towards the outer surface, which explains the different levels of sensitivity of different mycobacterial species to lipophilic inhibitors [28].

Based on the knowledge of valuable biological properties of rose essential oils, this study aimed to evaluate the antimycobacterial activity of the essential oils from Bulgarian oil-bearing roses *R. damascena* Mill., *R. alba* L., *R. centifolia* L., and *R. gallica* L. against *M. tuberculosis* H37Rv reference strain and clinical *M. tuberculosis* isolates representing the globally prevalent and medically significant Beijing and Latin-American Mediterranean (LAM) genotypes.

## 2. Materials and Methods

### 2.1. Rose Oil Distillation

**Figure 1:**
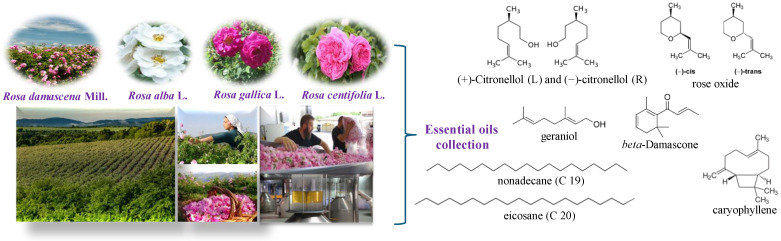
Collection of rose flowers and distillation of rose oil from *R. damascena* Mill., *R. alba* L., *R. centifolia* L., and *R. gallica* L. and some of their main compounds.

Fresh rose flowers of *R. damascena* Mill., *R. alba* L., *R. centifolia* L., and *R. gallica* L. were used as plant material (Figure 1). Roses were grown in the Experimental Field of the Rose Institute, Kazanlak, Bulgaria. The essential oil was obtained by traditional double distillation technology using a semi-industrial installation with a volume of 100 L.

### 2.2. Chemical Analysis: Gas Chromatography (GC-FID/MS)

The chemical composition of the rose oils was evaluated on an Agilent 7820A GC System coupled with a flame ionization detector and a 5977B MS detector (Santa Clara, CA, USA). The gas chromatographic analysis of rose oil. was done according to ISO 9842:2024 [29]. Two capillary columns were used: (i) non-polar EconoCapTM ECTM-5 (30 m × 0.32 mm × 0.25 µm) and (ii) polar HP-20M (50 m × 0.32 mm × 0.30 µm). Hydrogen (99.999%) was used as a carrier gas. The split ratio was 1:10, the inlet temperature was set to 250 °C, and the FID temperature was set to 300 °C. The non-polar column reveals a much richer spectrum of compounds and a better presentation of paraffin, but it is not suitable for dividing the main terpene alcohols citronellol and nerol since they have very similar retention times. For this reason, the polar column was used for better separation. The relative percentages of the components were calculated based on the GC peak areas without any correction factors. The oil constituents were identified by comparison of the retention indices (calculated using a standard calibration mixture of n-alkanes C8–C40 in n-hexane) and their mass spectra with those reported in the literature [30], as well as co-injections with authentic compounds.

### 2.3. M. tuberculosis Strains

The *M. tuberculosis* reference strain H37Rv was received from the Collection of the Scientific Center for the Expertise of Medicinal Products, Moscow, Russia (originally received from the Institute of Hygiene and Epidemiology, Prague, Czech Republic).

The *M. tuberculosis* clinical strains were recovered from the respiratory material of patients with pulmonary TB, permanent residents in St. Petersburg, Russia (strain 4542), and Ulan-Ude, Buryatia, Russia (strain 396). Strain isolation and identification and drug susceptibility testing of the strains were carried out using the automated system BACTEC MGIT 960. Strain 4542 was phenotypically resistant to isoniazid, rifampin, streptomycin, ethambutol, and pyrazinamide. It belongs to LAM SIT266. This spoligotype is known to be pre-XDR-associated, hypervirulent, epidemic in Belarus and found in Russia [31,32,33]. SIT266 belongs to Lineage 4 (or Euro-American lineage) and its sublineage 4.3.3 according to the SNP-barcode system [34], and in particular, to RD115 LAM-RUS clade. The other clinical isolate 396 was phenotypically resistant to isoniazid, rifampin, streptomycin, and ethionamide. It belongs to the Beijing 14717–15 cluster. It is endemic in Buryatia, Far East, Russia, and is MDR, hypervirulent, and highly lethal in the mice model [35,36]. Based on the SNP-barcode system it belongs to Lineage 2.1, characterized by intact region RD181, and belongs to the clade termed Beijing early ancient 1 [37] or Asia Ancestral 1 [38].

Finally, drug-susceptible *M. tuberculosis* reference strain H37Rv was used as a control. This strain belongs to Lineage 4 and its sublineage L4.9. More than one hundred years ago (when this strain was first isolated [39]) this lineage was widespread in the USA and Great Britain but currently has only a limited global dissemination.

### 2.4. Resazurin Microtitre Plate Assay

The REMA (resazurin microtitre plate assay) was performed as described previously [39]. A 3-week *M. tuberculosis* culture was transferred into a tube with glass beads, vortexed for 30 s and 5 mL Middlebrook 7H9 Broth (Becton Dickinson, Sparks, MD, USA) was added. The bacterial suspension was adjusted to 1.0 McFarland turbidity units (3 × 10^8^ bacteria/mL) and 20-fold diluted with Middlebrook 7H9 Broth with 10% OADC (Becton Dickinson). The same culture medium was used to prepare the 1:100 *M. tuberculosis* (1%) control. The stock solutions of the oil samples in DMSO (20 mg/mL) were diluted with Middlebrook 7H9 Broth (containing enrichment) to a concentration of 10 mg/mL. 200 μL of the obtained solution was introduced into the 3rd row of a 96-well microtitre plate. This row was used to perform 2-fold serial dilutions. Row 10 served as *M. tuberculosis* suspension control, row11—as 1% control (the same culture diluted 100-fold), and row 12—as a blank control for optical density reading (200 μL of the grown medium). The bacterial suspension (100 μL) was added into each well except rows 11 (1% control) and 12 (blank culture medium), to the total volume of 200 μL in each well. The plates were incubated at 35 °C for 7 days. At that point, 0.01% aqueous solution (30 mL) of resazurin (Sigma) was added to each well and the incubation continued for 18 h at 35 °C. The result was evaluated visually by comparing the color of the wells with isoniazid and test samples with the color of 1% control.

The REMA MIC determination was also performed for a known anti-TB drug (Isoniazid) to confirm that the condition used to determine MIC is appropriate (the tested concentrations ranged from 0.008–0.5 μg/mL). The REMA experiments were performed in the Mycobacteria reference laboratory at the St. Petersburg Institute of Phthisiopulmonology. The laboratory is externally quality assured by the System for External Quality Assessment “Center for External Quality Control of Clinical Laboratory Research” (Moscow, Russia). The REMA method was implemented in the laboratory within the frames of a multicenter European project coordinated by GSK-Spain, and was further shown reproducible also in comparison with other reference methods such as Bactec MGIT 960 system (Becton Dickinson, Sparks, MD, USA).

### 2.5. Molecular Analysis of Bacterial Strains and Bioinformatics

*M. tuberculosis* DNA extraction, molecular typing, including spoligotyping and detection of other molecular markers of the Beijing and LAM genotypes and subtypes was performed as described previously [33,36].

The bacterial DNA was submitted to whole genome sequencing on the Illumina HiSeq4000 platform. Whole genome, paired-end sequencing on the HiSeq4000 platform (Illumina, San Diego, CA, USA) was done using NEBNext Ultra, MiSeq Reagent v3, and PhiX Control v3 kits (Illumina, San Diego, CA, USA). DNA libraries were prepared using ultrasound DNA fragmentation and NEBNext Ultra DNA Library Prep Kit for Illumina (New England Biolabs, Ipswich, MA, USA). Data for the *M. tuberculosis* sequenced genomes were deposited in the NCBI Sequence Read Archive (strain 396–SRR18591741, strain 4542–SRR21776299).

SNP analysis of the WGS data was done using SAM-TB (https://samtb.uni-medica.com/index; accessed on 28 October 2024) and Phyresse (https://bioinf.fz-borstel.de/mchips/phyresse; accessed on 28 October 2024) online tools and manually confirmed using Geneious R package. The complete genome of strain H37Rv (NC_00962.3) was used as a reference for the alignment of short reads. The gene and protein information was taken from Mycobrowser online database (https://mycobrowser.epfl.ch/; accessed on 28 October 2024).

The role of the amino acid changes was evaluated using PAM1 (https://en.wikipedia.org/wiki/Point_accepted_mutation; accessed on 28 October 2024) and SIFT (https://sift.bii.a-star.edu.sg/www/SIFT_seq_submit2.html; accessed on 28 October 2024) using UniProtKB/Swiss-Prot and TrEMBL databases.

Multiple gene-gene interaction was assessed using the STRING tool at https://string-db.org/cgi/about.3 (accessed on 28 October 2024).

## 3. Results and Discussion

### 3.1. Chemical Composition and Antimycobacterial Activity of the Essential Oils from Rosa spp.

The chemical composition of the essential oils is presented in Table 1. The components are selected according to the last updated version of the international ISO 9842:2024 standard [29] for rose oil.

We further evaluated the antimycobacterial activity of the extracts from rose oil from four different *Rosa* species. MIC values of the four oil extracts were determined using a well-established REMA method while we tested MIC in the range from 0.04 to 5 mg/mL. A first-line anti-TB drug isoniazid served as a reference. MIC values were assessed for 3 different *M. tuberculosis* strains (1 reference strain and 2 clinical isolates). Based on spoligotyping and genotyping of the cluster-specific markers, the clinical isolates were assigned to the LAM family (spoligotype SIT266, LAM-RUS branch) and Beijing genotype (spoligotype SIT269, Asia Ancestral 2 branch).

The resulting MIC values for 4 oil samples against 3 strains are shown in Table 2.

The essential oil of *R. alba* showed the best antimycobacterial activity on the tested strains of different phylogenetic origins. Of the four oils tested, that of *R. gallica* showed the highest MIC values and, accordingly, the lowest activity, regardless of the tested strain. The essential oil of *R. centifolia*, similar to that of *R. alba*, showed the same activity against strain H37Rv, but significantly less inhibited the growth of the other two strains. The *R. damascena* oil showed intermediate MIC values compared to the other three samples (Table 2).

In our previous study, we determined the MIC values of the wastewater (WW) from the distillation of the four Bulgarian rose oils [40]. The MIC values were normalized to the content of total polyphenols in WW (mg/mL). Gram-positive *Staphylococcus aureus* was the most susceptible to inhibition, and we did not observe an antibacterial effect against *Pseudomonas aeruginosa*, *Escherichia coli*, and *Candida albicans*. WW from the distillation process of *R. alba* inhibited the proliferation of *S. aureus* by about 68%, WW from *R. damascena* by about 71%, WW from *R. centifolia* by 55% and WW from *R. gallica* by 64% at MIC. Interestingly, the most active effluent was from the distillation of *R. centifolia* oil (MIC 1.9 mg/mL). None of the WW showed a bactericidal or antifungal effect [40]. In the present study, the MIC values obtained for rose oils were much lower than those shown by WW against all other tested pathogens *S. aureus*, *P. aeruginosa*, *E. coli* and *C. albicans*.

Essential oils and the WW obtained from their distillation have a considerable difference in their chemical nature in terms of their hydrophilicity and lipophilicity. The ingredients of WW were found to contain large amounts of hydrophilic polyphenols, tannins and flavonoids in their forms such as aglycones and glycosides [40]. The essential oils of the four roses mainly contain lipophilic components (Table 1) and in these compositional differences we can also look for the difference in the manifested antimicrobial activities of the two types of biological extracts.

The antimicrobial activity of essential oils is due to their monoterpene alcohols including linalool, nerol, citronellol, and geraniol [41]. The antitubercular activity of citronellol, nerol, and geraniol against *M. tuberculosis* was previously evaluated in vitro and MIC values varied from 0.064 to 0.128 mg/mL ([41], and references therein); these MIC values obtained with pure compounds are expectedly lower than those observed in our study of the rose oil extracts.

A closer look at Table 1 and Table 2 suggests the most efficient *R. alba* oil extract is charac-terized by the highest relative percent of geraniol (30.98%) and nerol (10.92%). Specula-tively, the highest content of geraniol may be the key property behind the highest efficiency of the *R. alba* oil. In contrast, the highest level of citronellol was detected in the moderately efficient *R. damascena* oil (29.77%). On the other hand, the *R. gallica* oil was altogether the least efficient but it did not differ dramatically from all other oils in the individual com-ponents; for example, it has similarly low content of nerol as *R. centifolia* oil (4.72% and 4.36%, respectively). These findings considered together, it appears that the combined level of geraniol and nerol is a key factor that underlies the antimycobacterial action of the rose oil extracts. Further pharmacochemical research is required to understand the possibly synergistic interaction of different components of the studied essential oils.

It should be noted that MIC values of tested essential oils (0.16–0.31 mg/mL for the most promising samples) are much lower than MIC or critical concentrations for anti-tuberculosis drugs and chemical compounds that may potentially become anti-TB drugs that are in range of micrograms per mL [42,43]. However, the MIC of pure chemical compounds and MIC of essential oils cannot be compared directly.

In a recent study, Yuan et al. [44] tested rose oils on *Pseudomonas putida* and described their activity (MIC 10 mg/mL) as “excellent” compared to the thyme essential oil against the same bacterial species, 20 mg/mL, and MIC of *Enteromorpha* essential oil against *Bacillus cereus* (25 mg/mL) and *Staphylococcus aureus* (12.5 mg/mL). Cao et al. described the activity of different essential oils against *Mycobacterium abscessus*; some of the essential oils had MIC values of ~0.3 mg/mL and were termed as strongly active [45]. In another study, MIC of ginger essential oil against *M. tuberculosis* H37Rv was 0.125 mg/mL [46]. These values are similar to our findings on the most active rose oils (MIC 0.16–0.31 mg/mL). Furthermore, as noted above, the activity of the rose oils tested in our study was much higher against *M. tuberculosis* than against other bacteria such as *Staphylococcus aureus, Pseudomonas aeruginosa*, *Escherichia coli*, and *Candida albicans* [40]. In summary, we believe that our results show an overall good activity of some of these essential oils against *M. tuberculosis,* especially, *R. alba* essential oil (MIC 0.16–0.31 mg/mL). In turn, this implies that essential oil from *R. alba* may be further tested in the in vivo study, in particular, in the mouse model.

### 3.2. Antibacterial Action and Potentially Underlying Genomic Variation

Molecular mechanisms of antibacterial action of monoterpene alcohols from essential oils from plants and respectively, the genetics of bacterial resistance have been studied but remain obscure. For example, a study of *S. aureus* showed that menthone, a main component of peppermint oil, had a potent antibacterial effect on MRSA, and the mechanism of action involved the alteration of membrane structural components belonging to glycerophospholipids, glycolipids, and sphingolipids [47]. In *the M. tuberculosis* genome, multiple genes encode for these pathways (124 mutations in strain 396 and 80 mutations in strain 4542 in genes in the category Lipid metabolism. Since a total of 233 genes in this category were annotated in H37Rv [48]), an in silico analysis of SNP variation in these genes based solely on the available WGS data can hardly be an efficient approach. The gene deletion (in one but not another strain) can be a straightforward indication of the gene’s role in increased or reduced susceptibility, but such gene deletions are rare. It was shown that deletion of the *cyp138* gene (encoding for cytochrome P450 Cyp138) affected *M. tuberculosis* antibiotics susceptibility, and the levels of fatty acid metabolism, membrane-related proteins, and lipids such as triacylglycerol [49]. Interestingly, strain 396 (clinical strain relatively more susceptible to rose extracts in our study), had an in silico-assessed significant (PAM1 = 1) mutation in this gene in codon 192 Lys > Arg.

It should be noted that along with drug resistance i.e., significantly high MIC, there is a phenomenon of drug tolerance. This refers to strains that are resistant to low drug concentrations. For *M. tuberculosis*, its key responses to drug pressure specifically related to drug tolerance are reduced growth rates, metabolic shifting, and the promotion of efflux pump activity [50]. *M. tuberculosis* genome contains multiple genes related to the efflux/transport of different kinds of molecules such as inorganic ions, amino acids, peptides, polysaccharides, etc. Regarding pumps that efflux the terpenes in bacteria, a recent study of *Pseudomonas putida* analyzed its tolerance mechanisms to monoterpenoids (modified monoterpenes, containing oxygen functionality or missing a methyl group) and identified mutations in efflux pump promoter regions or associated transcription factors [51]. These authors also underlined that knowledge about tolerance mechanisms could allow a deeper insight into how bacteria can oppose monoterpenoid-containing drugs, like tea tree oil.

Since *M. tuberculosis* clinical strains did not differ dramatically in their MIC, we as-sumed that this difference could be due to variation in efflux genes that may account for *M. tuberculosis* drug tolerance. We further hypothesized that some in silico significant mutations specific for the most susceptible (to rose extracts) strain 396, negatively affect protein structure and account for this situation. We used the available WGS data and looked at differences between the clinical MDR *M. tuberculosis* strains in their efflux pump genes. We checked mutations in the studied clinical strains in the complete list of the efflux pump genes [52] and the complete list of mutations in these genes in strains 396 and 4542 is shown in Table S1. The significance of the nonsynonymous mutations was assessed using PAM 1 and SIFT (Table 3).

There were only 2 nonsynonymous mutations in 2 efflux genes in LAM strain 4542 (Rv0194 74 M > T and mmpL5 948 I > V) and, interestingly, both were present in strain 396. Phylogenetically, these two clinical strains are very distant, and finding of the same mutations (Rv0194 74M > T and mmpL5 948I > V) in different genes in these strains implies their value for strain survival on a long-term time scale even if SIFT P value is non-significant. Another explanation is that it is reference strain H37Rv that had “mutations”, compared to the ancestral alleles in strains 396 and 4542 and these mutations could lead to its in-creased susceptibility. It should be kept in mind that H37Rv belongs to evolutionarily younger lineage L4.9 compared to ancestral sublineage of the Beijing genotype (strain 396) and in this sense it is not ideal reference genome. Indeed, this was the first sequenced M. tuberculosis strain in 1997 and historically it became a reference for genomic comparisons. Speculatively, it may be that these alleles in H37Rv render it the most susceptible to most of the tested rose oils compared to the two clinical MDR strains. It is also tempting to hy-pothesize that these two clinical MDR strains have developed during their evolution un-der selective pressure of different anti-TB drugs drug tolerance mechanisms that also permitted them to gain a certain level of intrinsic tolerance to the essential oils.

Beijing strain 396 had 10 additional nonsynonymous mutations in 9 genes (Table 3). Given the lower MIC in strain 396, we hypothesized that such numerous mutations in these genes could be deleterious and thus related to weaker efflux and increased susceptibility of this strain to rose essential oils. In total, two mutations in strain 396 were concordantly significant by both PAM1 and SIFT methods of the in silico analysis: Rv0194 1098 P-L and Rv2688c 213 C-R. These concordantly significant potentially deleterious mutations in Rv0194 1098 P-L and Rv2688c 213 C-R could affect (reduce) efflux in strain 396 hence its increased susceptibility and double-lower MIC. *Rv2688c* encodes for antibiotic ABC transporter ATP-binding protein and *Rv0194* encodes for multidrug ABC transporter ATPase/permease i.e., these two proteins belong to the same family of ATP-binding cassette (ABC) transporters [53] and may interact epistatically.

**Figure 2:**
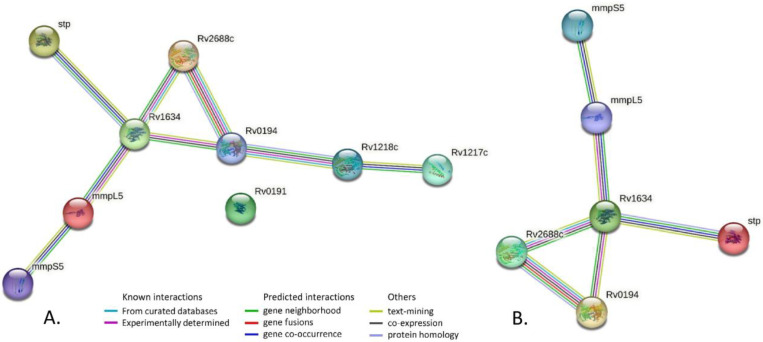
Gene-gene network analysis. (**A**). All 9 efflux genes with mutations in strain 396. PPI enrichment *p*-value = 9.52 × 10^−8^ i.e., the network has significantly more interactions than expected. (**B**). Six efflux genes with significant mutations in strain 396. PPI enrichment *p*-value: 1.36 × 10^−6^.

Gene-gene network analysis of all 9 efflux genes with mutations in strain 396 (Table 2) revealed multiple interactions (perhaps not surprising since these genes all belong to the same gene category Cell wall and cell processes) (Figure 2A). Gene-gene network analysis of only 6 efflux genes *Rv0194*, *mmpL5*, *mmpS5*, *Rv1634*, *Rv2688c*, *Rv2333c* that harbored significant mutations in strain 396 (marked in bold in Table 2) showed a strong interaction (Figure 2B). Given strong interaction of these 6 genes, furthermore supported by experimental studies, it may be that possible deleterious mutations detected in strain 396 could act synergistically and reduce its efflux pump efficiency hence lower MIC for the tested oil extracts.

On the whole, it would be too speculative to link presence of the particular alleles in the above genes, MIC of the studied strains by different essential oils, and content of these oils. It appears that mutations in efflux genes in both clinical strains make them more re-sistant to most of the tested oils compared to H37Rv reference strain. The *R. gallica* oil is an exception but this oil was otherwise the least active.

## 4. Conclusions

To conclude, the antimycobacterial activity of the essential oil extracts from different *Rosa* spp. was tested on the reference and clinical strains of *M. tuberculosis*. *R. alba* oil extract was the most promising when tested on all *M. tuberculosis* strains of different phylogenetic backgrounds. On the other hand, *R. gallica* was the least active. Our findings on the chemical composition of the studied oil extracts and their MIC for different strains considered together, it appears that the combined (and high) level of geraniol and nerol is a key factor that underlies antimycobacterial action of the rose oil extracts. In this view, geraniol and nerol, along with isoniazid, may be considered as additional positive controls in the MIC testing of rose essential oils against *M. tuberculosis* strains.

Placed in the context of similar studies of the antibacterial activity of the essential oils from plants, our results demonstrate an overall good activity of some of the rose essential oils against *M. tuberculosis,* especially, *R. alba* essential oil (MIC 0.16–0.31 mg/mL). In turn, this implies that essential oil from *R. alba* may be reasonably considered for further in vivo study in the mouse model. An aerosol inhalation or microcapsules were previously tested for essential oils from different plants and pathogenic bacteria in the mouse model of infection [54]. In the future, a similar approach may be pursued for *R. alba* essential oil which was found the most active against different *M. tuberculosis* strains in our study.

Two clinical MDR strains have likely developed during their previous adaptation to anti-TB drugs certain drug tolerance mechanisms that also permitted them to demonstrate intrinsic tolerance to the essential oils. Our results show heterogeneity of rose oil action on different mycobacterial strains. This highlights the importance of considering the real genetic diversity of the clinical isolates when assessing the antimicrobial efficiency of new compounds and natural products. It is critically important to analyze such efficiency with clinically and epidemically relevant strains either those highly transmissible and/or multidrug-resistant and globally spread. A further experimental study of the *R. alba* oil antimycobacterial properties (also in combination with key anti-TB drugs) and mycobacterial resistance/susceptibility genomics is warranted on the large panel of the genetically and geographically diverse clinical *M. tuberculosis* strains.

Essential oils cannot replace the standard antibacterial chemotherapy but may complement it. It is known that efflux pump inhibitors are useful supplements to the antibiotics providing a synergistic action against different bacteria [55]. Geraniol is an active constituent of *Helichrysum italicum* essential oil, and it was shown to significantly increase the efficacy of β-lactams, quinolones, and chloramphenicol by targeting the efflux pump mechanism [21,56]. The 2.5% essential oil of *H. italicum* was found to significantly reduce chloramphenicol resistance in *Enterobacter aerogenes*, *E. coli*, *P. aeruginosa*, and *Acinetobacter baumannii*. Combinations of this essential oil with phenylalanine arginine β-naphthylamide showed synergistic activity. Lorenzi et al. concluded that this essential oil contains an agent with the activity of an efflux pump inhibitor [56]. Thus, Bulgarian rose essential oils can increase *M. tuberculosis* susceptibility to anti-TB drugs by inhibiting bacterial efflux pumps. Further studies are warranted to investigate a possible synergistic action of the new-generation anti-TB drugs and the most promising rose oil extracts on the large panel of different bacterial strains with known overexpression of efflux pumps.

Lastly, we note that TB patients frequently suffer from mental disorders because of prolonged treatment, side effects of anti-TB drugs and relapses of the disease [57,58,59,60]. Thus, in light of their excellent aromatic properties, low toxicological profile and ease of administration, rose oils could serve as a complementary or non-pharmacological modality to relieve anxiety, alleviate stress and depressive states, promote relaxation, optimize cognitive function of such patients.

## Figures and Tables

**Table 1:** The chemical composition of the essential oils (main and character constituents in rel. %).

Compound	RI Calc.	RI Lit.	*R. damascena*	*R. alba*	*R. gallica*	*R. centifolia*
Ethanol	489	489	0.09 ± 0.02	0.10± 0.01	0.01 ± 0.00	0.01 ± 0.00
Phenylethanol	1110	1110	0.48 ± 0.02	0.22 ± 0.02	0.29 ± 0.03	0.10 ± 0.01
**Citronellol**	1229	1228	29.77 ± 0.42	12.64 ± 0.92	8.38 ± 0.20	7.60 ± 0.22
**Nerol**	1751 *	1751 *	11.04 ± 0.12	10.92 ± 0.70	4.72 ± 0.52	4.36 ± 0.42
**Geraniol**	1248	1246	21.05 ± 0.90	30.98 ± 0.10	21.93 ± 0.04	15.35 ± 0.12
Methyl eugenol	1405	1405	1.30 ± 0.10	0.56 ± 0.04	0.94 ± 0.33	0.62 ± 0.10
Heptadecane (C_17_)	1700	1700	1.64 ± 0.00	1.98 ± 0.07	2.78 ± 0.10	1.38 ± 0.09
Nonadecene(C_19:1_)	1880	1880	2.30 ± 0.14	4.41 ± 0.24	1.71 ± 0.07	1.75 ± 0.02
Nonadecane (C_19_)	1901	1901	11.12 ± 0.06	11.70 ± 0.07	21.18 ± 0.10	18.62 ± 0.10
Eicosane (C_20_)	2000	2000	1.08 ± 0.05	1.21 ± 0.00	1.60 ± 0.12	0.96 ± 0.02
Heneicosane (C_21_)	2100	2100	7.02 ± 0.72	9.86 ± 0.09	9.07 ± 0.12	6.78 ± 0.04

Note. RI—Retention index. *—RI of nerol applies to a HP-20M column. All others refer to EconoCapTM ECTM-5 column. The key monoterpene alcohols (Citronellol, Nerol, Geraniol) are in bold.

**Table 2:** MIC values for rose oil samples tested on *M. tuberculosis* strains (mg/mL).

Essential Oils	H37Rv (Susceptible, L4.9)	4542 (MDR, LAM Genotype)	396 (MDR, Beijing Genotype)
*R. alba* L.	0.16	0.31	0.31
*R. centifolia* L.	0.16	1.25	0.62
*R. damascena* Mill.	0.31	1.25	0.62
*R. gallica* L.	1.25	1.25	0.62

MDR—multidrug-resistant; LAM—Latin-American Mediterranean.

**Table 3:** Nonsynonymous mutations in efflux genes in strain Beijing 396 (relatively more susceptible to the rose oil extracts).

Genome Pos.	Label	Gene	Codon	aa	PAM1 **	SIFT *P* **	Protein Function (Based on Information from Micobrowser and STRING Tool)
222925	*Rv0191*	*Rv0191*	213	A-T	22	0.11	MFS-type transporter. Probable conserved integral membrane protein; Active efflux pump that plays an important role in chloramphenicol resistance. Overexpression causes pyrazinamide resistance
227098 *	*Rv0194*	*Rv0194*	74	M-T	**6**	1.00	Multidrug ABC transporter ATPase/permease Probable transmembrane multidrug efflux pump; Overexpression in M. smegmatis increases resistance to erythromycin, ampicillin, novobiocin, and vancomycin.
230170	*Rv0194*	*Rv0194*	1098	P-L	**3**	**0.00**	
775639 *	*Rv0676c*	*mmpL5*	948	I-V	57	1.00	Probable conserved transmembrane transport protein mmpl5; Part of an export system, which is required for biosynthesis and secretion of siderophores
776100	*Rv0676c*	*mmpL5*	794	T-I	**7**	1.00	
778743	*Rv0677c*	*mmpS5*	55	V-M	**4**	0.08	Possible conserved membrane protein mmps5; Part of an export system, which is required for biosynthesis and secretion of siderophores. Essential for virulence
1361285	*Rv1217c*	*Rv1217c*	173	A-T	22	0.18	Probable tetronasin-transport integral membrane protein ABC transporter; Probably part of the ABC transporter complex *Rv1217c-Rv1218c* involved in the resistance to a wide range of structurally unrelated drugs. Probably responsible for the translocation of the substrate across the membrane
1362006	*Rv1218c*	*Rv1218c*	243	Q-R	10	0.60	Probable tetronasin-transport atp-binding protein ABC transporter; Probably part of the ABC transporter complex *Rv1217c-Rv1218c* involved in the resistance to a wide range of structurally unrelated drugs. Could be involved in the efflux of substrates belonging to the diverse chemical classes of novobiocins, biarylpiperazines, pyridines, bisanilinopyrimidines, pyrroles and, to a smaller extent, pyrazolones. Probably responsible for energy coupling to the transport system
1839759	*Rv1634*	*Rv1634*	198	G-R	**0**	0.52	Possible drug efflux membrane protein; Could be involved in fluoroquinolones efflux
3005014	*Rv2688c*	*Rv2688c*	213	C-R	**1**	0.00	Antibiotic-transport ATP-binding protein ABC transporter; Part of the ABC transporter complex *Rv2686c/Rv2687c/Rv2688c* involved in fluoroquinolones export. Probably responsible for energy coupling to the transport system
3005185	*Rv2688c*	*Rv2688c*	156	P-T	**5**	0.83	
2608117	*Rv2333c/Stp*	*Rv2333c/Stp*	69	D-Y	**3**	1.00	Integral membrane drug efflux protein stp; Contributes to spectinomycin and tetracycline resistance

* This mutation was also found in strain LAM 4542. ** Significant SIFT *p* (<0.05) and low PAM1 (<10) are in bold.

## Data Availability

The data supporting the conclusions of this article are presented in the main text and Supplementary Materials.

## Supplementary Materials

The following supporting information can be downloaded at: https://www.mdpi.com/article/10.3390/pharmaceutics16111393/s1, Table S1. Complete list of mutations in the efflux pump genes in strains 396 and 4542. Figure S1. Gas chromatography (GC-FID/MS) analysis of the essential oils from different *Rosa* species.
