# Supplementary material for: Antimycobacterial Activity of Essential Oils from Bulgarian Rosa Species Against Phylogenomically Different Mycobacterium tuberculosis Strains
Source: Pharmaceutics. 2024 Oct 29;16(11):1393. doi: 10.3390/pharmaceutics16111393 (PMC11597806; doi:10.3390/pharmaceutics16111393)
Supplement: Supplementary file 1 [file pharmaceutics-16-01393-s001.zip › pharmaceutics-3174462-supplementary/Fig. S1. Rose oils GC analysis.pdf]

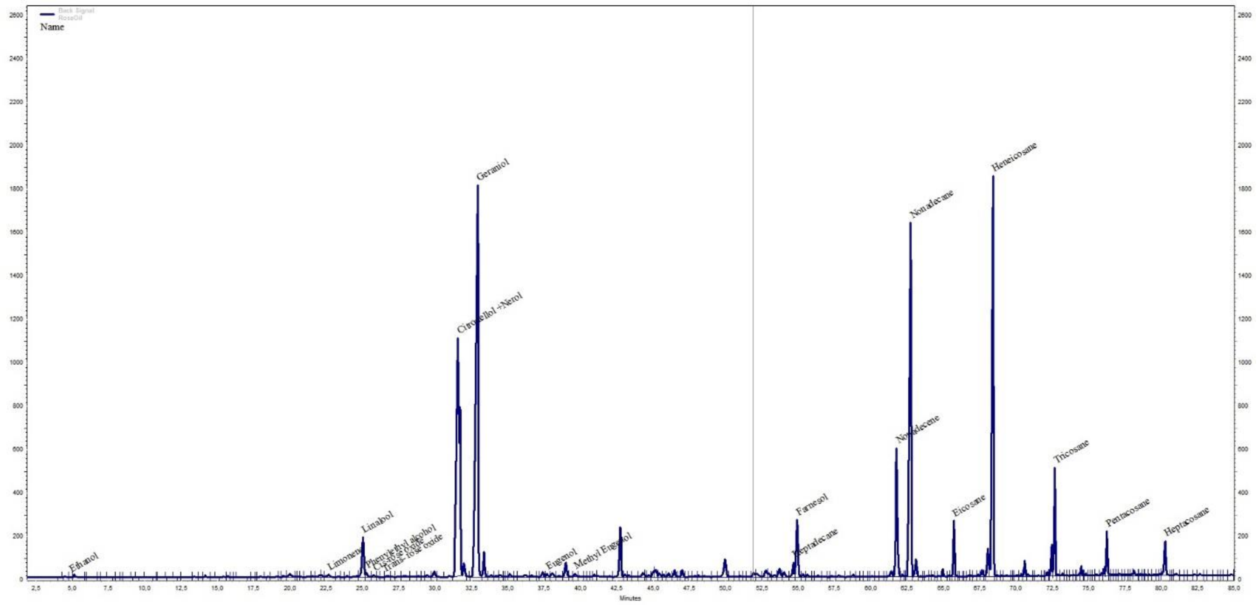

R. alba

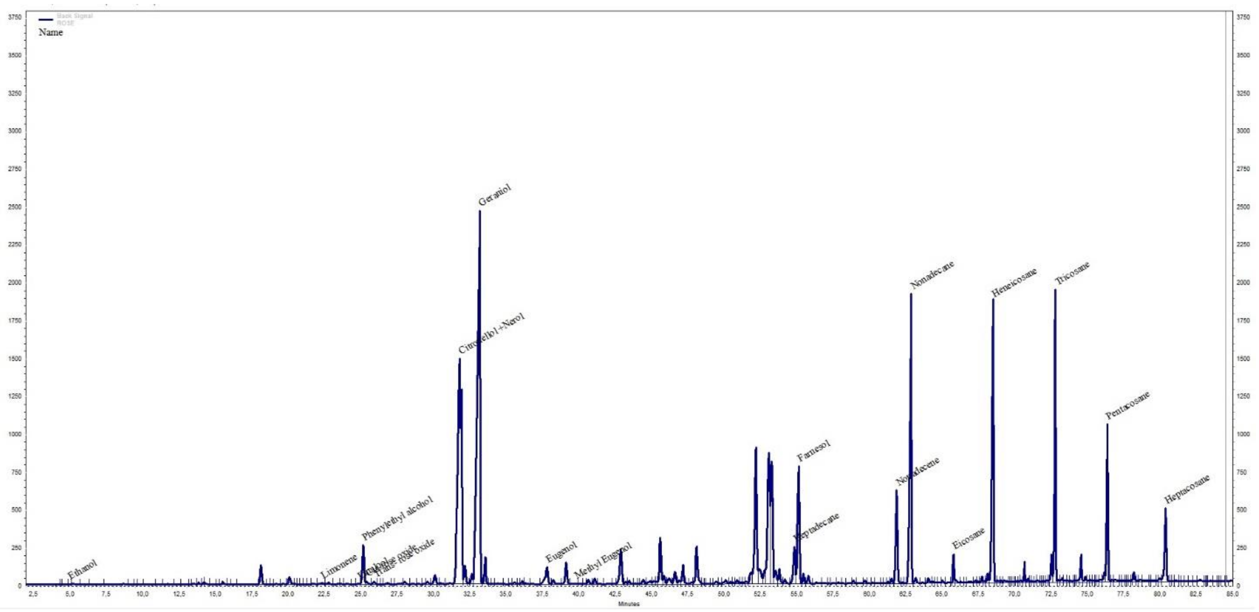

R. centifolia

Figure S1. Gas chromatography (GC-FID/MS) analysis of the essential oils from different *Rosa* species.

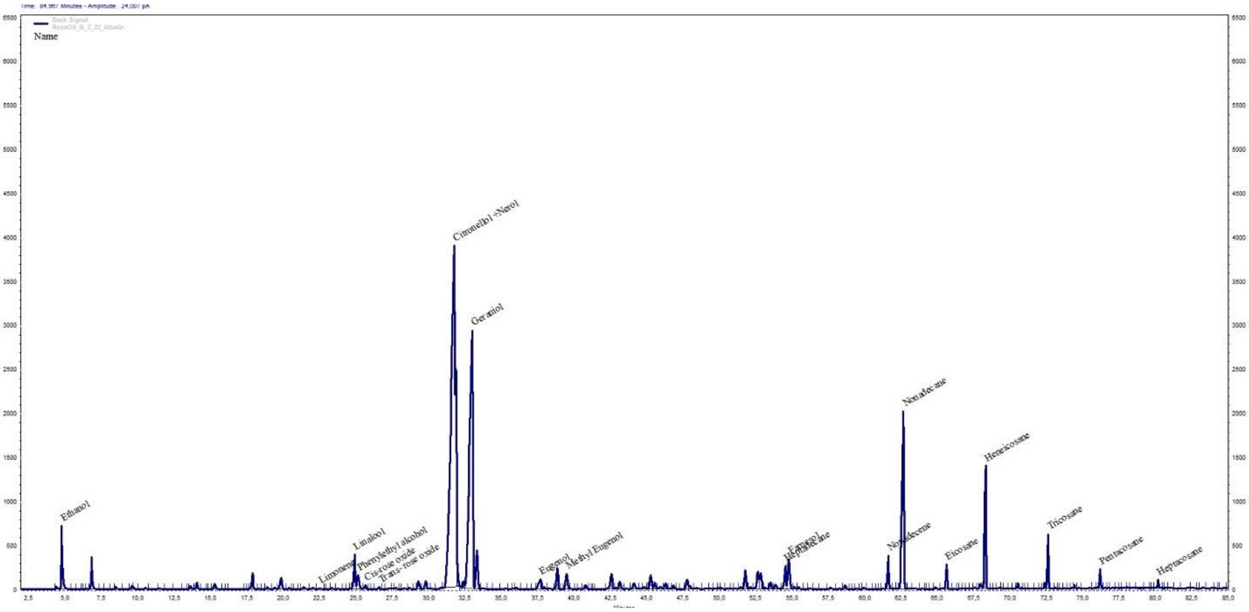

R. damascena

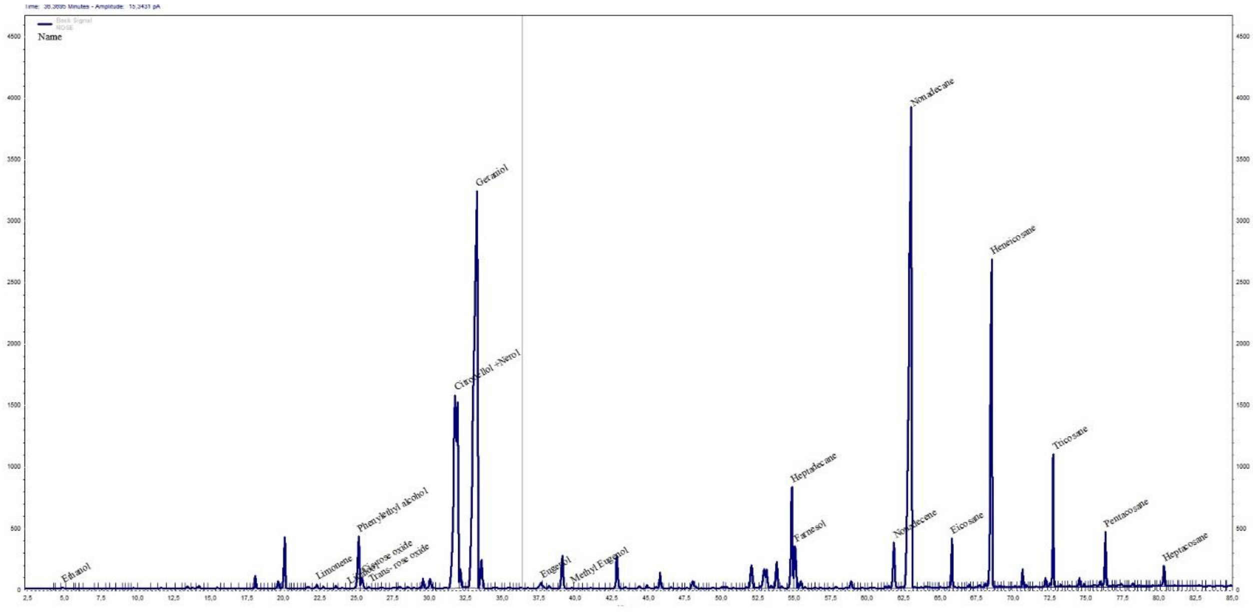

R. gallica

Figure S1. Gas chromatography (GC-FID/MS) analysis of the essential oils from different *Rosa* species.
