# Supplementary material for: Antimycobacterial Activity of Essential Oils from Bulgarian Rosa Species Against Phylogenomically Different Mycobacterium tuberculosis Strains
Source: Pharmaceutics. 2024 Oct 29;16(11):1393. doi: 10.3390/pharmaceutics16111393 (PMC11597806; doi:10.3390/pharmaceutics16111393)
Supplement: Supplementary file 1 [file pharmaceutics-16-01393-s001.zip › pharmaceutics-3174462-supplementary/Table S1 - R1.pdf]

Table S1. Mutations in efflux pump genes identified in clinical *M. tuberculosis* strains 396 and 4542.

SNPs were detected in WGS data and annotated using SAM-TB online tool (https://samtb.uni-medica.com/index). The list of efflux pump genes was taken from Laws et al. (2022).

(A) strain 396.

position in genome	codon_	type	amino acid change	codon (nt) change	label in H37RV	gene	**efflux pump gene** (Laws et al., 2022)	function	class	gene position	type of change
222925	213	Nonsyn	A-T	GCT-ACT	Rv0191	Rv0191	**Rv0191**	MFS-type transporter	Cell wall and processes	637	SNP
227098	74	Nonsyn	M-T	ATG-ACG	Rv0194	Rv0194	**Rv0194**	multidrug ABC transporter ATPase/permease	Cell wall and processes	221	SNP
230170	1098	Nonsyn	P-L	CCC-CTC	Rv0194	Rv0194		multidrug ABC transporter ATPase/permease	Cell wall and processes	3293	SNP
775639	948	Nonsyn	I-V	ATT-GTT	Rv0676c	mmpL5	**Rv0676c/77c**	transmembrane transport protein MmpL5	Cell wall and processes	2842	SNP
776100	794	Nonsyn	T-I	ACC-ATC	Rv0676c	mmpL5		transmembrane transport protein MmpL5	Cell wall and processes	2381	SNP
778743	55	Nonsyn	V-M	GTG-ATG	Rv0677c	mmpS5		membrane protein MmpS5	Cell wall and processes	163	SNP
876141		Intergenic		G-T	Rv0780-Rv0783c	purC-emrB	**Rv0783c/EmrB**	phosphoribosylaminoimidazole-succinocarboxamide synthase / multidrug resistance protein EmrB	Intermediary metabolism and respiration /Cell wall and processes	--	SNP

938240	43	Synon	P-P	CCG-CCC	Rv0842	Rv0842	**Rv0842**	integral membrane protein	Cell wall and processes	129	SNP
1360209	531	Synon	A-A	GCA-GCG	Rv1217c	Rv1217c	**Rv1217c/18c**	tetronasin ABC transporter integral membrane protein	Cell wall and processes	1593	SNP
1361190	204	Synon	S-S	AGC-AGT	Rv1217c	Rv1217c		tetronasin ABC transporter integral membrane protein	Cell wall and processes	612	SNP
1361285	173	Nonsyn	A-T	GCT-ACT	Rv1217c	Rv1217c		tetronasin ABC transporter integral membrane protein	Cell wall and processes	517	SNP
1362006	243	Nonsyn	Q-R	CAA-CGA	Rv1218c	Rv1218c		tetronasin ABC transporter ATP-binding protein	Cell wall and processes	728	SNP
1839759	198	Nonsyn	G-R	GGG-CGG	Rv1634	Rv1634	**Rv1634**	multidrug-efflux transporter	Cell wall and processes	592	SNP
1894300		Insert		ins_GTCTTGCCGC	Rv1666c/Rv1669	cyp139/Rv1669	**Rv1667c/68c**	cytochrome P450 Cyp139/hypothetical protein Rv1669	Intermediary metabolism and respiration/Conserved hypotheticals	-738/-1425	INSERTION
2608117	69	Nonsyn	D-Y	GAC-TAC	Rv2333c (stp)		**Rv2333c (stp)**	Integral membrane drug efflux protein Stp	Cell wall and cell processes	205	SNP
1894422		Intergenic		A-G	Rv1666c/Rv1669	cyp139/Rv1669	**Rv1666c/Rv1669**	cytochrome P450 Cyp139/hypothetical protein Rv1669	Intermediary metabolism and respiration/Conserved hypotheticals	-860/-1303	SNP
3005014	213	Nonsyn	C-R	TGC-CGC	Rv2688c	Rv2688c	**Rv2686c/87c/88c**	antibiotic ABC transporter ATP-binding protein	Cell wall and processes	637	SNP
3005185	156	Nonsyn	P-T	CCC-ACC	Rv2688c	Rv2688c	**Rv2686c/87c/88c**	antibiotic ABC transporter ATP-binding protein	Cell wall and processes		SNP
3614982	874	Synon	L-L	CTA-CTG	Rv3239c	Rv3239c	**Rv3239c**	transmembrane transport protein	Cell wall and processes	2622	SNP

(B) strain 4542

**genome pos**	**codon _pos**	**type**	amino acid change	codon (nt) change	**label in H37Rv**	**gene**	**efflux pump gene** (Laws et al., 2022)	**function**	**class**	**gene position**	type of change
227098	74	Nonsynonymous	M-T	ATG-ACG	Rv0194	Rv0194	**Rv0194**	multidrug ABC transporter ATPase/permease	Cell wall and processes	221	SNP
775639	948	Nonsynonymous	I-V	ATT-GTT	Rv0676c	mmpL5	**Rv0676c/77c**	transmembrane transport protein MmpL5	Cell wall and processes	2842	SNP
1360209	531	Synonymous	A-A	GCA-GCG	Rv1217c	Rv1217c	**Rv1217c/18c**	tetronasin ABC transporter integral membrane protein	Cell wall and processes	1593	SNP
1586249	506	Synonymous	Q-Q	CAG-CAA	Rv1410c	Rv1410c	Rv1410c	aminoglycosides/tetracycline-transport integral membrane protein	Cell wall and processes	1518	SNP
3614982	874	Synonymous	L-L	CTA-CTG	Rv3239c	Rv3239c	**Rv3239c**	transmembrane transport protein	Cell wall and processes	2622	SNP

References

Laws M, Jin P, Rahman KM. Efflux pumps in Mycobacterium tuberculosis and their inhibition to tackle antimicrobial resistance. Trends Microbiol. 2022 Jan;30(1):57-68. doi: 10.1016/j.tim.2021.05.001.
